# Long-lived cancer-resistant rodents as new model species for cancer research

**DOI:** 10.3389/fgene.2012.00319

**Published:** 2013-01-09

**Authors:** Jorge Azpurua, Andrei Seluanov

**Affiliations:** Department of Biology, University of RochesterRochester, NY, USA

**Keywords:** aging, cancer, naked mole rat, blind mole rat, long-lived rodents

## Abstract

Most rodents are small and short-lived, but several lineages have independently evolved long lifespans without a concomitant increase in body-mass. Most notable are the two subterranean species naked mole rat (NMR) and blind mole rat (BMR) which have maximum lifespans of 32 and 21 years, respectively. The longevity of these species has sparked interest in the tumor suppression strategies that may have also evolved, because for many rodent species (including mice, rats, guinea pigs, gerbils, and hamsters) tumors are a major source of late-life mortality. Here, we review the recent literature on anti-cancer mechanisms in long-lived rodents. Both NMR and BMR seem to have developed tumor defenses that rely on extra-cellular signals. However, while the NMR relies on a form of contact inhibition to suppress growth, the BMR evolved a mechanism mediated by the release of interferon, and rapid necrotic cell death. Although both organisms ultimately rely on canonical downstream tumor suppressors (pRB and p53) the studies reveal species can evolve different strategies to achieve tumor-resistance. Importantly, studies of these cancer-resistant rodents may benefit human health if such mechanisms can be activated in human cells.

Mice have become the preferred model for cancer research due to the presence of a powerful arsenal of molecular and genetic tools, their short generation time, strain variety, and propensity for neoplasia. A major goal of cancer research is to understand the genetic and molecular changes that underlie transformation and what defense mechanisms fail as people grow older or are exposed to oncogenic insults. Due to the extreme tumor propensity of mice, focusing research solely in this species may miss some important tumor suppression mechanisms used by longer-lived animals.

Some important differences at the molecular level between mouse and human cells have already been identified. The tumor suppression profile of mice is markedly different from that of human cells, with the most immediate distinction being the presence of telomerase activity in somatic tissue (Gorbunova and Seluanov, 2009). Mouse fibroblasts require fewer mutations to transform than human cells; elimination of pRB and p53 signaling coupled with constitutive Ras signaling is sufficient for mice whereas humans require the activation of telomerase as well as mutations that prevent dampening of the AKT signaling pathway (such as PP2A or PTEN) (Hahn and Weinberg, 2002; Rangarajan and Weinberg, 2003). The three products of the *INK4* tumor suppression locus, p15^INK4b^, p16^INK4a^, and ARF, contribute differently to the tumor-resistance of human and mice, with ARF loss being much more deleterious to mice than humans (Kim and Sharpless, 2006).

To find novel tumor suppressive mechanisms that could potentially be applied to human treatments, we chose to study organisms that have a tumor-resistance profile similar to that of humans, but are tractable to research and investigation in ways similar to mice. Furthermore, by investigating animals that are phylogenetically related to Murinae, we could ask questions about the evolution of tumor suppression mechanisms (and longevity in general). New results from non-model rodents show that there are still gaps in our understanding of anti-cancer mechanisms. Currently, we are investigating two such non-standard model rodent species: the naked mole rat (*Heterocephalus glaber*, hereafter referred to as “NMR”) and the blind mole rat (*Spalax sp*. or *Nannospalax sp*., hereafter referred to as “BMR”) (Figure 1).

**Figure 1:**
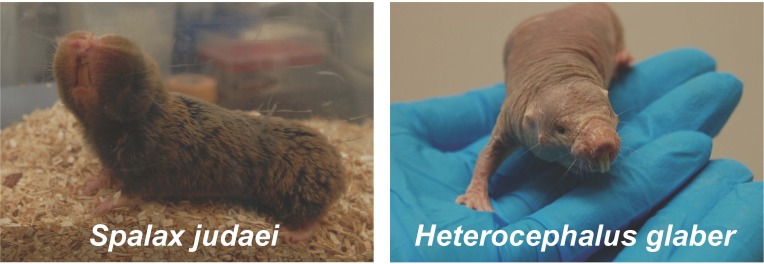
**Images of naked mole rat (right) and blind mole rat (left)**.

*H. glaber* has been of interest to a wide range of biologists due to its unusual life history traits and adaptations for a subterranean ecological niche. It is considered to be a eusocial rodent, with only one breeding female per colony (and is consequently a naturally highly inbred animal), and has a maximum lifespan of more than 30 years (Buffenstein and Jarvis, 2002; Buffenstein, 2005, 2008) making it an outlier on the body-mass/longevity plot at the same order as *H. sapiens*. Furthermore, it has a much lower incidence of neoplasia than traditional inbred laboratory mice, with no observed tumors from various laboratories housing thousands of the rodents (Edrey et al., 2011).

The NMR has already started yielding interesting observations regarding its tumor-resistance, despite being a relatively new experimental animal. In a 2009 paper by Seluanov et al., we showed that NMR fibroblasts grow much more slowly in tissue culture than other rodent cells, and halt their cell cycle at much lower cell densities than other rodents (a phenomenon termed early contact inhibition, or ECI) (Seluanov et al., 2009). NMRs were also shown to be highly resistant to induced tumorigenesis: primary fibroblasts could not be transformed (induced into anchorage independent growth) solely by disrupting p53 and pRB in the presence of oncogenic Ras signaling, the cells had to undergo additional mutations during passaging in tissue culture to allow transformation. The nature of the mutations was not established, but disruption of p16^INK4a^ was observed, as well as a loss of the ECI phenotype. Therefore, although loss of ECI in tissue culture was not sufficient for transformation, it was a necessary step before the cells could undergo anchorage independent growth in soft agar.

The tumor-resistance of NMR cells has also been investigated *in vivo* by Liang et al. by injection of NMR cells with various combinations of transforming factors into immunocompromised mice (Liang et al., 2010). Again, knockout of the p53 and pRB pathways by LargeT antigen was not sufficient to induce tumorigenesis, even in the presence of a constitutive Ras oncogenic protein, while these mutations were sufficient to allow mouse cells to form large tumor masses in the immunocompromised mice. Tumor formation was observed, however, by addition of hTERT in addition to these other factors. While NMR fibroblasts express their own telomerase (Seluanov et al., 2007; Gomes et al., 2011) and can be passaged indefinitely in tissue culture, the additional pro-growth targets of hTERT (Rahman et al., 2005; Lee et al., 2008) may be sufficient to induce tumor formation when present with other transforming factors.

The BMR shows a similar longevity and tumor-resistance (de Magalhaes and Costa, 2009; Nasser et al., 2009) to the NMR despite being phylogenetically more related to mice. They are also long-lived and subterranean, although they are solitary and genetically heterogeneous. In our recent study Gorbunova et al. showed that in tissue culture, BMR fibroblasts displayed a novel phenotype which we named concerted cell death (CCD) (Gorbunova et al., 2012). Here, fibroblasts grow normally for several population doublings before undergoing synchronized rapid cell death. Like the NMR, the BMR also has somatic telomerase expression, which eliminated a telomere attrition-based response as the culprit. We showed that in both growing and dying cells, the telomeres were still long and telomerase was still active.

Because the death of cells was synchronized, we hypothesized that during growth, a signaling factor was being secreted that would kill cells upon reaching a threshold concentration. The authors identified interferon beta (IFN-β) as the secreted factor that was increasingly released by the cells into the media during passage in tissue culture. Freshly isolated primary BMR cells treated with media conditioned by BMR cells that were near CCD were rapidly induced to undergo CCD themselves. It was also possible to induce very high levels of apoptosis in mouse lines (but not human lines, possibly due to divergence in the structure of the IFN-β receptor). We interpreted the secretion of IFN-β as a response to rapid growth in tissue culture, reflecting the sensitivity of the cells to over-proliferation or abnormalities in the local microenvironment. Intriguingly, the BMR evolved this p53-dependent mechanism despite undergoing a mutation in its p53 gene as an adaptation to hypoxia that renders it less capable of directly promoting apoptosis (Ashur-Fabian et al., 2004). Presumably, the other targets of p53 are sufficient to kill the cell in the presence of IFN-β.

The BMR CCD and the NMR ECI responses are markedly different in their phenotype (cell death vs. growth arrest) and show how convergent evolution toward tumor-resistance can take different paths. Nonetheless, there are some important similarities. In both cases, the authors show that elimination of both the pRB and the p53 pathways is important. If either tumor suppression pathway is left intact, the cells will still undergo arrest or death. This is one trait which these long-lived rodents share more with humans than mice. Additionally, in both NMR and BMR an extra-cellular signal is mediating some aspect of the tumor-resistance (cell density and interferon response, respectively), suggesting that in long-lived species with somatic telomerase activity, selection favors increased cellular sensitivity to the external environment.

During the evolution of extreme longevity in rodents, selection for tumor-resistance seems to be tremendously important (Figure 2). Comparative studies in rodents are yielding novel insights into the evolution of tumor suppressor mechanisms, which seem to arise sporadically rather than being a conserved basal trait of the rodent lineage. Interestingly, each lineage evolves its own tumor suppression mechanism, which means there may more to learn from other small, long-lived rodents, such as the Eastern gray squirrel, chinchilla, or muskrat. By learning about alternative tumor suppression mechanisms evolving in different lineages, novel targets for anti-cancer therapy may be revealed or important cell cycle regulatory circuits hitherto ignored may be discovered.

**Figure 2:**
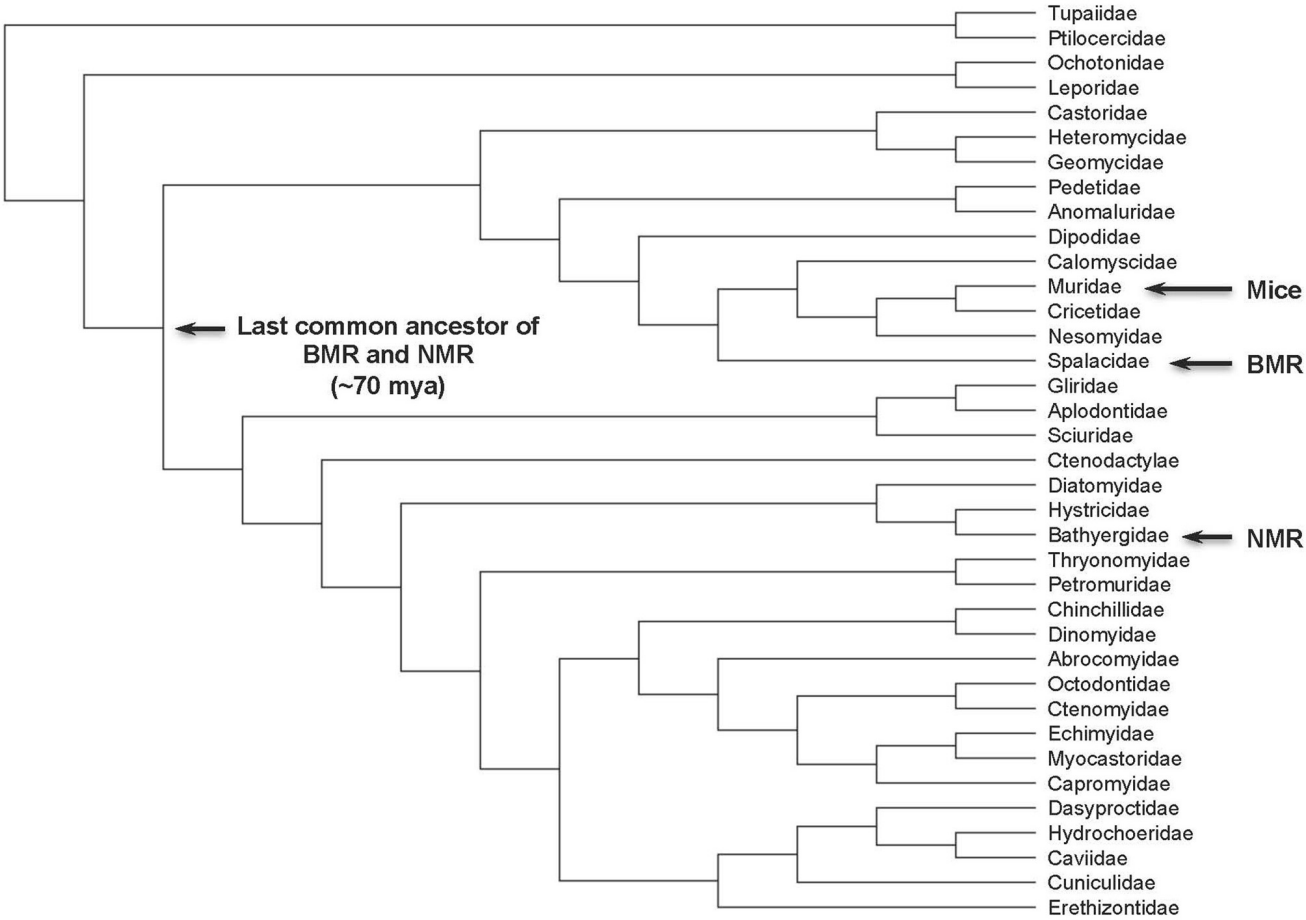
**Rodent phylogeny**. The positions of naked mole rat, blind mole rat, and mouse are indicated. The tree topology is adapted from Meredith et al. (2011).

## Conflict of interest statement

The authors declare that the research was conducted in the absence of any commercial or financial relationships that could be construed as a potential conflict of interest.
